# Antimicrobial Efficacy of Silver-based Formulations against *Fusobacterium nucleatum* : A Mini-scoping Review

**DOI:** 10.1055/s-0045-1814460

**Published:** 2026-01-20

**Authors:** Nabiha Remmani, Ayaana Kamal, Nabeel Humood Alsabeeha, Iman Kamal, Hien Chi Ngo, Kausar Sadia Fakhruddin, Sameh S.M. Soliman, Hiroshi Egusa

**Affiliations:** 1 College of Dental Medicine, University of Sharjah, Sharjah, United Arab Emirates; 2 Division of Molecular and Regenerative Prosthodontics, Tohoku University Graduate School of Dentistry, Sendai, Japan; 3 Faculty of Medicine, Georgian National University SEU, Georgia; 4 Department of Restorative Dentistry, Ajman University, Ajman, United Arab Emirates; 5 UWA Dental School, The University of Western Australia, Nedlands, Western Australia, Australia; 6 College of Pharmacy, University of Sharjah, Sharjah, United Arab Emirates

**Keywords:** *Fusobacterium nucleatum*, silver nanoparticles, antimicrobial efficacy, periodontal pathogens, cytotoxicity, glutathione-stabilized silver

## Abstract

*Fusobacterium nucleatum* is a critical periodontal pathogen implicated in biofilm maturation and inflammation in periodontitis. With growing concerns over antibiotic resistance, silver-based antimicrobial agents are emerging as promising alternatives. This minireview aims to evaluate the antimicrobial efficacy and biocompatibility of various silver formulations, including silver nitrate (AgNO _3_ ), silver diamine fluoride, silver nanoparticles (AgNPs), and glutathione-stabilized AgNPs (GSH-silver), specifically against *F. nucleatum* . A systematic literature search (1990–2024) across PubMed, EMBASE, EBSCOhost, and Google Scholar identified five *in vitro* studies. Key outcome measures included minimum inhibitory concentration (MIC), cytotoxicity, and proinflammatory responses. All formulations demonstrated significant antimicrobial efficacy, with 5 nm AgNPs showing the most potent effect (MIC = 25 µg/mL). Silver(I) carbohydrate complexes and GSH-silver showed enhanced efficacy and reduced cytotoxicity. However, elevated proinflammatory cytokine release was noted with some formulations. Silver-based formulations exhibit potent antimicrobial activity against *F. nucleatum* , but their inflammatory potential and cytotoxicity warrant further investigation. Future *in vivo* studies are needed to optimize dosing, assess tissue interactions, and validate the clinical safety of these silver-based formulations.

## Introduction

Biofilms are resilient and dynamic, substratum-attached microbial networks prevalent in nature, particularly in the oral cavity, where over 700 microbial species form multispecies communities on tooth surfaces and in supragingival and subgingival niches. 1 Upon maturation, biofilms develop a well-defined structure that protects resident microbes from antimicrobial agents and host immunity, enabling survival in fluctuating, challenging environments. 2 Early colonizers, such as *streptococci and Actinomyces* , initially adhere to host oral and dental surfaces, followed by co-adhesion and co-aggregation with other microbial species, leading to biofilm maturation. 1 2 *F. nucleatum* , a Gram-negative anaerobe, plays a crucial role in this process and facilitates the formation of mature biofilms. 3 *Fusobacterium* species and subspecies impact the composition and architecture of supragingival and subgingival biofilms differently. 3 Residing in the anaerobic periodontal pocket, *F. nucleatum* promotes the adherence and integration of other periodontal microbes, such as *Porphyromonas gingivalis* , *Treponema denticola* , and red and orange complex bacteria and fungi, thereby contributing to the structural integrity of the subgingival polymicrobial biofilms. 3

The periopathogen *F. nucleatum* has multiple virulence attributes, including adhesins that facilitate attachment to host tissues and other bacteria, hemolytic activity, and hydrogen sulfide production. 4 It also stimulates the production of matrix metalloproteinases (MMPs) by host cells, leading to tissue degradation and loss of periodontal attachment. 4 5 These factors contribute to its ability to disrupt host immune responses and induce inflammation. 6

Interactions between bacteria and immune cells are particularly noteworthy. Additionally, *F. nucleatum* exhibits several mechanisms to evade host immune defenses, which pose a significant challenge in combating this pathogen. 7 It modulates the immune response by inhibiting the activity of neutrophils and other phagocytic cells, reducing their ability to kill the bacteria. 7 8 This immune evasion enables *F. nucleatum* to persist within periodontal pockets, contributing to chronic inflammation and tissue destruction. 9

Thus, *F. nucleatum* plays a multifaceted role in periodontitis, involving intricate interactions within the subgingival biofilm, immune evasion, virulence attributes, and its function as a bridging organism that enhances the stability and pathogenicity of the biofilm. This makes it a formidable periodontal pathogen. 10 The management of periodontal disease typically involves mechanical debridement, such as scaling and root planning, often supplemented with local or systemic antibiotics. 11 However, the persistent nature of periodontal pathogens and the rise of antibiotic-resistant strains pose significant treatment challenges. These limitations highlight the need for alternative antimicrobial agents to enhance treatment outcomes and reduce disease recurrence. 11

Silver, recognized for its broad-spectrum antimicrobial properties, has been used in various medical applications, including wound dressings and coatings for medical devices. 12 Its antimicrobial effects are attributed to its ability to disrupt microbial cell membranes, interfere with enzyme functions, and induce oxidative stress within microbial cells. 12

Silver-based formulations, including silver diamine fluoride (SDF), silver nanoparticles (AgNPs), and silver nitrate (AgNO _3_ ), are used to manage dental conditions such as caries and dentine hypersensitivity. 13 Additionally, SDF has been used successfully to reduce gingival inflammation and manage hyperplastic gingivitis. 14 15 Several studies have demonstrated the antibacterial activity of silver-based compounds against various oral pathogens, including *F. nucleatum* , as adjunct treatments in periodontal therapy. 16 17 Recent advances in nanomaterials have refined silver's clinical potential beyond its traditional antibacterial scope. Newer nanoscale composites and silver–organic hybrids demonstrate not only enhanced antimicrobial efficacy but also improved biocompatibility. For instance, reviewed controlled-release silver dressings minimize cytotoxicity, while sustaining antimicrobial ion flux. 18 19 20 21

However, a gap exists in reviews specifically addressing silver formulations and *F. nucleatum* . In this systematic review, we aimed to collate studies detailing silver formulations tested as antimicrobials against *F. nucleatum* , including their potential and limitations.

## Methods

### Data Sources

A scoping systematic review was conducted using the PubMed, Google Scholar, EMBASE, and EBSCOhost databases to identify relevant English-language RCTs, *in vitro* and ex vivo studies from peer-reviewed journals.

#### Search Terms

The following search terms were used to select qualifying studies from the databases, covering articles published between January 1, 1990 and January 31, 2024: (“ *In Vitro* Techniques”[MeSH] AND “Periodontitis/microbiology”[MeSH]) AND (“Silver”[MeSH] OR “Silver Nitrate”[MeSH] OR “Silver Nanoparticles”[MeSH] OR “Silver Diamine Fluoride”[MeSH]) AND (“Microbial Sensitivity Tests”[MeSH] OR “Colony Count, Microbial”[MeSH] OR “Minimum Inhibitory Concentration”[MeSH])

“Antimicrobial activity of silver against periodontal pathogens” AND “Silver nitrate” AND “Periodontitis”

(“ *in vitro* study” AND “Periodontal pathogen”) AND (“Silver nitrate” OR “Silver nanoparticle” OR “Silver diamine fluoride”) AND (“Minimum inhibitory concentration” OR “Bacterial colony count” OR “Antibacterial efficacy”)

(“ *In vitro* assay” AND “Periodontal pathogens”) AND (“Silver compounds” OR “Silver nitrate antimicrobial” OR “Silver nanoparticles antibacterial” OR “Silver diamine fluoride periodontitis”) AND (“Microbial sensitivity” OR “Minimum inhibitory concentration” OR “Antibacterial activity” OR “Cytotoxicity and inflammation assays”).

#### Focused Question

What is the antimicrobial efficacy of various silver-based formulations against *Fusobacterium nucleatum* , and what are their potential advantages and limitations in periodontal therapy?

### Outcome Measure

To evaluate and summarize the antimicrobial efficacy of different silver-based formulations (e.g., silver nitrate, silver diamine fluoride, silver nanoparticles, and silver(I) carbohydrate complexes) against *Fusobacterium nucleatum* .

### Study Selection

#### Inclusion Criteria

Studies were included if they met the following criteria:

(a) Clinical trials, *in vitro* , *in vivo* , *and ex vivo* studies assessing the antimicrobial efficacy of silver-based compounds against periodontal pathogens, specifically *F. nucleatum* .
(b) Detailed interventions specifying concentrations and methods of application.
(c) Quantitative data on antimicrobial efficacy (e.g., minimum inhibitory concentration [MIC] values, CFU reduction).
(d) Qualitative data on cytotoxicity and inflammatory responses, if available.
(e) Peer-reviewed journal articles.
(f) Articles published in English.

#### Exclusion Criteria

Studies were excluded if they met any of the following criteria:

(a) Studies assessing the antimicrobial efficacy of silver-based compounds against periodontal pathogens other than *F. nucleatum* .
(b) Studies that did not align with the predetermined study objectives or were limited to abstracts, reviews, or commentaries.
(c) Studies with unspecified or unclear intervention methods or concentrations.
(d) Studies lacking quantitative measures of antimicrobial efficacy.

### Data Search and Data Analysis

**Fig. 1:**
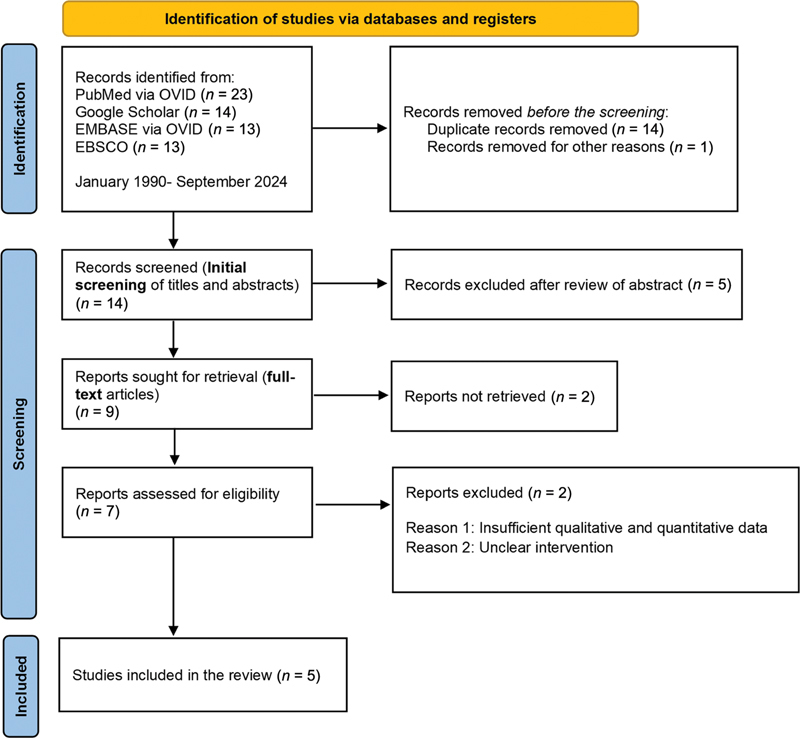
PRISMA flowchart outlining the literature search process and study selection.

We adhered to the Preferred Reporting Items for Systematic Reviews and Meta-Analyses for Scoping Reviews (PRISMA-ScR) guidelines to ensure a systematic approach. 22 Fig. 1 illustrates the search strategy and the outcomes. A meta-analysis was not feasible due to the heterogeneity in silver compound formulations and concentrations, complicating data pooling for quantitative analysis.

During the initial phase of our three-step electronic data search and analysis, we (N.B., K.S.F., A.K., and I.K.) assessed the titles and abstracts of relevant studies to identify those meeting the predefined inclusion criteria. In the second phase, a detailed examination of the full texts of the identified articles was conducted by the reviewers (A.K. and I.K.) to gain a comprehensive understanding of the data. This rigorous analysis ensured that the selected studies met the eligibility criteria and that the reported outcomes aligned with our objectives. Additionally, a backward search was performed by K.S.F. and N.B., scrutinizing the references of the included studies to identify any other relevant research.

In the final phase, the reviewers (N.B. and A.K.) extracted and evaluated data from the selected studies. The specific characteristics of each included study, such as study setting, samples, intervention, and country, were recorded using the Cochrane model. Other factors, such as sample size, evaluation time, assessment methods, and study conclusions, were systematically examined to effectively synthesize the findings.

For managing the identified manuscripts, bibliographic software EndNote version 12 (Clarivate Analytics, USA) was used. Table 1 summarizes the characteristics of the included *in vitro* trials and their findings regarding the antimicrobial efficacy of silver-based formulations against *F. nucleatum* . This table provides a concise overview of the key findings of each study. Table 2 presents a quantitative cross-study comparison, consolidating numerical data and threshold values to illustrate the heterogeneity observed in MIC ranges, nanoparticle dimensions, and cytotoxicity outcomes.

**Table 1:** Key characteristics of the reviewed studies

Study Year Country	Study design	Samples (microbes)	Intervention details	Mechanism of action	Study outcome
**Spacciapoli et al** 2001 USA	An *in vitro* assay	Bacterial strains, including *Porphyromonas gingivalis, Prevotella intermedia, P. denticola, Bacteroides forsythus, Fusobacterium nucleatum vincentii, Campylobacter gracilis, C. rectus, Eikenella corrodens, Actinobacillus actinomycetemcomitans* , and several *Streptococcus* species	Metal ions ( **silver nitrate** , copper chloride, and zinc chloride) were tested. Concentrations of silver nitrate ranging from 0.5 to 50 mg/mL. Assays were conducted in phosphate-buffered saline (PBS) and human serum, mimicking conditions in the periodontal pocket.	Silver's antimicrobial activity might result from its ability to bind to the essential enzyme sulfhydryl, disrupting bacterial function.	Silver nitrate exhibited strong antimicrobial activity against all tested periodontal pathogens at a 0.5 mg/mL concentration, reducing at least 3 log _10_ in CFU/mL.
**Zhong Lu et al** 2013 China	An *in vitro* assay	**Anaerobic oral bacteria:***Streptococcus mutans, S. sanguis, S. mitis, Actinobacillus actinomycetemcomitans,* and *Fusobacterium nucleatum.* **Aerobic bacterium:** *E. coli*	**Silver nanoparticles (Ag NPs)** with mean sizes of ∼5, 15, and 55 nm were synthesized using different methods (reduction and hydrothermal methods). **Testing methods:** ***Colony counting assay*:** bacterial colonies after exposure to Ag NPs. ***Growth inhibition curve method:*** determine the minimum inhibitory concentration (MIC) of Ag NPs.	Ag NP's possible attachment to bacterial cell membranes disrupts membrane permeability. Possible penetration of the inner membrane, inactivating respiratory chain dehydrogenases. The nano-size possibly results in a larger surface area, increasing interaction with bacterial cells.	Ag NPs showed significant antibacterial activity. The antibacterial activity of Ag NPs was size-dependent, with smaller particles (5 nm) exhibiting the highest antibacterial activity. The MIC values for 5-nm Ag NPs against *F. nucleatum* was 25 µg/mL.
**Rams et al** 2020 USA	An *in vitro* assay	Freshly isolated red and orange complex periodontal pathogens from subgingival biofilm samples from 24 adults with severe periodontitis.	**Silver diamine fluoride (SDF—38% and 19%)** Total viable counts. Specific periodontal pathogens were quantified using established phenotypic criteria and MALDI-TOF mass spectrometry. Dilution aliquots of each subgingival specimen were mixed with either 38 or 19% SDF and inoculated onto enriched Brucella blood agar plates, followed by anaerobic incubation for 7 days at 37°C. Control samples without SDF exposure.	The silver component in SDF exerts pronounced antimicrobial activity.	About 87.5% of subgingival samples were culture-negative for periodontal pathogens after exposure to either 38 or 19% SDF. Both 38 and 19% SDF significantly reduced total subgingival viable counts compared with controls ( *p* < 0.001). There were no significant differences in antimicrobial effects between 38 and 19% SDF.
**Zorraquín-Peña et al** 2020 Spain	An *in vitro* assay	*Porphyromonas gingivalis, Fusobacterium nucleatum* , *Streptococcus mutans*	**Glutathione-stabilized silver nanoparticles (GSH-AgNPs)** ranged from 3.08 to 98.50 µg/mL in the concentrations. *Cytotoxicity assay:* MTT assay *Inflammatory response:* ELISA *silver accumulation:* Inductively coupled plasma mass spectrometry (ICP-MS).	Two main hypotheses: A. Direct interaction with the cell membrane B. Release of ionic silver	GSH-AgNPs demonstrated significant antibacterial activity against key periodontal pathogens, with a manageable cytotoxic and inflammatory response in oral cells at lower concentrations.
**Reise et al** 2016 Germany	An *in vitro* assay	*Fusobacterium nucleatum, Aggregatibacter actinomycetemcomitans, Porphyromonas gingivalis, Streptococcus mutans, Enterococcus faecalis.*	**Silver(I) carbohydrate complexes (3 and 4)** ** Silver nitrate (AgNO _3_ ) ** MIC and agar diffusion assays at 10 and 20 mM concentrations. Cytocompatibility assay using live/dead staining of human gingival fibroblasts	Silver ion interaction with bacterial DNA. Destruction of cell membranes. Blocking of essential enzymes, disrupting electron transport.	Silver (I) carbohydrate complexes showed greater antibacterial efficiency than AgNO _3_ and demonstrated lower cytotoxicity on human gingival fibroblasts.

**Table 2:** Comparative summary of minimum inhibitory concentrations (MICs), nanoparticle sizes, and cytotoxicity thresholds reported in studies evaluating silver-based formulations against *Fusobacterium nucleatum*

Study Year	Silver formulation	Particle size/form	MIC (µg/mL or mM)	Tested cell type	Non-toxic/ Cytotoxic concentration	Cell viability (%) at safe dose	Key remarks
**Spacciapoli et al** 2001	AgNO _3_ (metallic salt)	Ionic form	500 µg/mL	NA	NA	NA	Broad antibacterial activity but no cytotoxicity data reported.
**Zhong Lu et al** 2013	AgNPs	5 nm, 15 nm, 55 nm	25 µg/mL (5 nm)	HGF	≤25 µg/mL	≈80%	Antibacterial efficacy size-dependent; smaller NPs = greater potency.
**Reise et al** 2016	Ag(I) carbohydrate complex vs. AgNO _3_	Molecular complex	10–20 mM	HGF	≤10 mM	>85%	10× less cytotoxic than AgNO _3_ ; enhanced solubility.
**Rams et al** 2020	Silver diamine fluoride 19 and 38%	Ionic solution	NA	NA	0.01% = cytotoxic (literature)	30–40% (HGF, Ho 2022)	Strong antibacterial but high fibroblast toxicity; used in multispecies biofilm.
**Zorraquín-Peña et al** 2020	GSH-stabilized AgNPs	5–10 nm (stabilized)	24.63 µg/mL	HGF	≤6.16 µg/mL	>90%	Potent antibacterial; transient cytokine upregulation (IL-6, IL-8, TNF-α).

Abbreviations: HGF, human gingival fibroblast; NA, not available.

#### Quality and Overall Risk of Bias Assessment of the Included Reports

Two researchers (A.K. and I.K.) independently assessed the quality of the reports included and the overall risk of bias. In case of discrepancies, the third and fourth reviewers (N.B. and K.S.F., respectively) were consulted to reach a consensus. The quality of the selected studies was evaluated individually using the Quality Assessment Tool for In Vitro Studies (QUIN Tool). 23 This evaluation considered 12 criteria: clearly stated aims/objectives, detailed sample size calculation, comprehensive explanation of sampling techniques, information on comparison groups, detailed methodology, operator details, randomization, outcome measurement methods, outcome assessor details, blinding, statistical analysis, and result presentation. Each criterion was scored as follows: adequately specified (score = 2), not adequately specified (score = 1), not specified (score = 0), or not applicable (NA). The final score for each study was calculated by adding the scores of the applicable criteria. This score was then used to categorize studies as high (<50%), medium (50 to 70%), or low risk (>70%) of bias. The risk of bias was calculated using the formula: Final score = (Total score × 100)/(2 × number of applicable criteria) ( Table 3 ). Studies receiving >70% of total possible points were classified as “low risk,” reflecting complete methodological reporting (for instance, Spacciapoli et al, Reise et al). Scores between 50 and 70% indicated “moderate risk,” often due to partial omission of operator randomization or blinding (for example, Zorraquín-Peña et al). None scored <50%, indicating generally acceptable methodological rigor.

**Table 3:** Risk of bias assessment

Study	Clearly stated aims/objectives	Detailed explanation of sample size calculation and sampling technique	Details of comparison group	Detailed methodology, operator details and randomization	Method of measurement of outcome	Outcome assessor detail, blinding	Statistical analysis	Presentation of results	SCORE	BIAS
**Spacciapoli et al, 2001**	2	1	2	2	2	0	1	2	12	L
**Zhong Lu et al, 2013**	2	1	2	2	2	0	1	1	11	L
**Reise et al, 2016**	2	1	2	2	2	0	1	2	12	L
**Rams et al, 2020**	2	1	2	1	2	0	1	2	11	L
**Zorraquín-Peña et al, 2020**	2	1	1	2	1	0	1	1	9	M

Abbreviation: NA, not applicable.

Notes: Score: adequately specified: 2 points; inadequately specified: 1 point; not specified: 0 points. Bias: low risk (L) > 70%; medium risk (M) between 70 and 50%; high risk (H) < 50%.

## Results

The reviewed studies 24 25 26 27 28 evaluated the antimicrobial efficacy of various silver-based compounds against periodontal pathogens. All studies employed an *in vitro* assay design to test the effectiveness of metallic silver salts 24 26 27 and silver nanoparticles 25 28 against different strains of bacteria relevant to periodontal diseases.

### Antimicrobial Efficacy of Metallic Silver Salts Against *F. Nucleatum*

In 2001, Spacciapoli et al tested metal ions such as silver nitrate, copper chloride, and zinc chloride, at concentrations ranging from 0.5 to 50 mg/mL. 24 These experiments, conducted in phosphate-buffered saline and human serum to mimic periodontal pocket conditions, revealed that silver nitrate exhibited strong antimicrobial activity against all tested periodontal pathogens, including *F. nucleatum* , at a concentration of 0.5 mg/mL, achieving at least a 3 log10 reduction in CFU/mL. The authors suggested that the antimicrobial activity of silver might be due to its ability to bind to essential enzyme sulfhydryl groups, thereby disrupting bacterial functions. Over a decade later, Reise et al (in 2016) 26 evaluated silver(I) carbohydrate complexes and silver nitrate using MIC and agar diffusion assays at concentrations of 10 and 20 mM. Their observations indicated that silver(I) carbohydrate complexes were more effective than AgNO _3_ . The antimicrobial effects of silver were attributed to its interactions with bacterial DNA, cell membrane destruction, and inhibition of essential enzymes. Recently, Rams et al 27 tested SDF at concentrations of 38 and 19% on freshly isolated subgingival biofilm samples from adults with severe periodontitis. The results revealed that 87.5% of the subgingival samples were culture-negative for periodontal pathogens following exposure to either 38 or 19% SDF, with both concentrations significantly reducing total viable counts compared with the controls. Furthermore, the study by Rams et al was the only study to test multispecies biofilms, underscoring the need for future multispecies models.

### Antimicrobial Efficacy of Silver Nano-particles Formulations Against *F. Nucleatum*

Zhong Lu et al 25 synthesized silver nanoparticles with mean sizes of approximately 5, 15, and 55 nm using reduction and hydrothermal methods. The antibacterial efficacy of these nanoparticles was assessed by colony counting and determination of the minimum inhibitory concentration, which revealed significant activity. Notably, the smallest particles (5 nm) exhibited the highest efficacy, with an MIC value of 25 µg/mL against *F. nucleatum* . The researchers suggested that Ag-NPs attach to bacterial cell membranes, disrupt membrane permeability, potentially penetrate inner membranes, and inactivate respiratory chain dehydrogenases. Additionally, the increased surface area of the smaller nanoparticles enhances their interaction with bacterial cells.

Another study by Zorraquín-Peña et al 28 investigated glutathione-stabilized silver nanoparticles (GSH-Ag-NPs) at Ag ^+^ ion concentrations ranging from 3.08 to 98.50 µg/mL. The results demonstrated significant antibacterial activity of GSH-Ag-NPs against *F. nucleatum* . However, doses higher than 24.63 µg/mL were required to achieve a significant inhibition ( *p* < 0.01) of *F. nucleatum* growth.

### Cytotoxicity and Inflammatory Responses of the Silver Formulations on Periodontal Tissue

Zorraquín-Peña et al 28 investigated the cytotoxicity of glutathione-stabilized silver nanoparticles on a human gingival fibroblast cell line using MTT assay, at Ag concentrations of 6.16, 12.31, and 24.63 µg/mL after 30 minutes and 24 hours of exposure. At a concentration of 6.16 µg/mL, GSH-Ag-NPs did not exhibit significant cytotoxicity, maintaining cell viability above 90%. However, cell viability decreased substantially at concentrations of 12.31 and 24.63 µg/mL, with reductions of 25 and 40%, respectively, compared with control cells.

Similarly, Reise et al 26 evaluated the cytocompatibility of silver(I) carbohydrate complexes and AgNO _3_ on human gingival fibroblasts. AgNO _3_ demonstrated cytotoxic effects at concentrations exceeding 0.02 mM. In contrast, the silver complex tris[2-(β-D-thio-glucopyranosyl) ethyl]-amine-silver(I)-nitrate (complex 3) exhibited a significantly reduced cytotoxicity, being approximately 10 times less toxic to human gingival fibroblasts compared with AgNO _3_ .

### Inflammatory Response of Silver-based Formulations

Zorraquín-Peña et al exclusively studied the inflammatory response induced by glutathione-stabilized silver nanoparticles by measuring the production of proinflammatory cytokines IL-6, IL-8, and tumor necrosis factor-α (TNF-α) using ELISA after 30 minutes and 24 hours of exposure. 28 GSH-AgNPs caused upregulation of IL-6, IL-8, and TNF-α expression compared with control cells, with higher levels observed after 24 hours than after 30 minutes of exposure. Notably, IL-8 production was significantly higher than IL-6 and TNF-α. The highest IL-6 production occurred at a concentration of 6.16 µg/mL, while IL-8 and TNF-α release peaked at 3.08 µg/mL. At concentrations affecting cell viability (≥12.31 µg/mL), an inverse relationship was observed between cytokine production and cell viability.

## Discussion

The results from the reviewed studies collectively demonstrate that silver-based formulations, both in nano- and non-nano sizes, exhibit significant antimicrobial activity against periodontal pathogens, including *F. nucleatum* . However, different formulations, including silver nitrate, silver nanoparticles, silver diamine fluoride, and silver(I) carbohydrate complexes, each present unique advantages and challenges in managing periodontal infections. To the best of our knowledge, this is the first comprehensive literature review examining the antimicrobial efficacy of silver formulations against *F. nucleatum* specifically.

Zhong Lu et al 25 investigated the antimicrobial efficacy of silver nanoparticles, with a particular emphasis on their size and concentration. Their findings underscored the significance of nanoparticle size in enhancing antibacterial activity. This increased potency is primarily attributed to the smaller nanoparticles' superior ability to penetrate bacterial cells more efficiently and their larger surface area for interaction. 29 These properties enable silver nanoparticles to disrupt bacterial cells more effectively, inhibiting critical metabolic and genetic functions and thereby reducing bacterial growth. 29

**Fig. 2:**
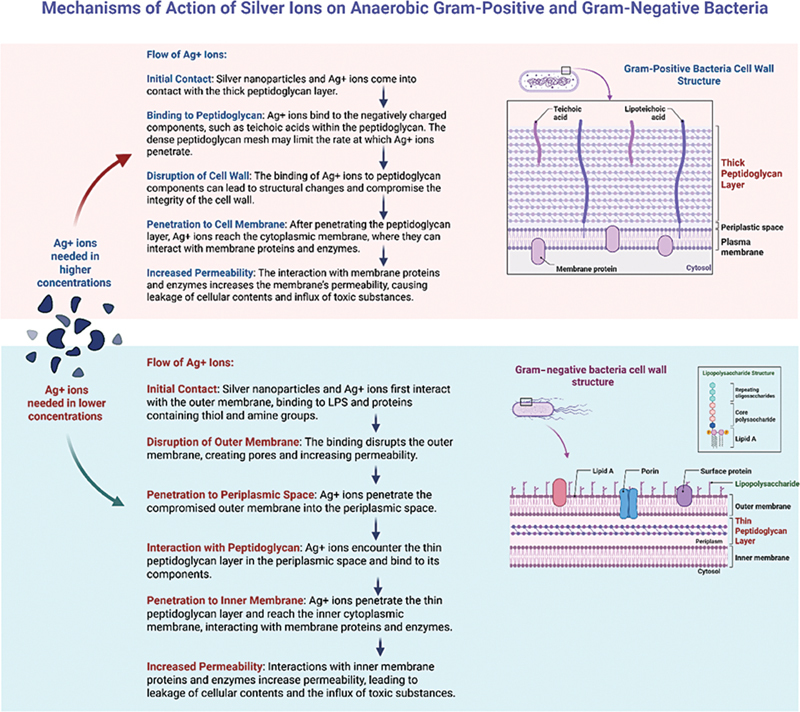
Comparison of silver ion concentrations required for membrane disruption in Gram-positive and Gram-negative bacteria.

In the case of *F. nucleatum* and *A. actinomycetemcomitans* , both Gram-negative anaerobic bacteria, the observed MIC was 25 µg/mL. This is lower than the 50 µg/mL required for Gram-positive *Streptococcus* species. The difference in MICs can be attributed to variations in the cell wall structures. Gram-negative bacteria possess a thin peptidoglycan layer, an outer membrane containing proteins with thiol and amine groups and negatively charged lipopolysaccharides. 30 These structural components possibly enhance the permeability of the bacterial cell wall to silver ions, facilitating more effective antimicrobial action. 30 In contrast, Gram-positive bacteria have thicker peptidoglycan layers, which require higher doses of silver ions for effective penetration and binding. 30 Thus, the structural differences between Gram-negative and Gram-positive bacteria significantly influence their susceptibility to silver nanoparticle treatment. Gram-negative bacteria, with their thinner cell walls and accessible outer membranes, are more susceptible to the antimicrobial effects of nano-sized silver particles, possibly resulting in a lower MIC for these organisms ( Fig. 2 ).

Our systematic review highlights the promising role of glutathione (GSH)-stabilized silver nanoparticles in periodontal therapy due to their multifaceted benefits. GSH, a potent antioxidant, plays a crucial role in mitigating the cytotoxic effects induced by nano-silver particles. 31 This protection occurs through the binding of silver components to the thiol (-SH) group present in GSH, which neutralizes the free silver ions released upon application. This interaction not only stabilizes silver nanoparticles at the nanoscale but also reduces their reactivity, minimizing potential cellular damage and inflammatory responses. 31

Furthermore, the structural stabilization provided by GSH helps prevent silver from penetrating membranous surfaces, which could otherwise lead to weakened mitochondrial membranes, electron leakage, and increased production of reactive oxygen species (ROS). 31 32 Additionally, GSH's antioxidant properties are beneficial for managing oxidative stress within periodontal tissues. By scavenging ROS, such as hydrogen peroxide (H _2_ O _2_ ) and superoxide (O _2_^−^ ), GSH helps counteract oxidative damage, thereby preserving cellular integrity. 31 33 This mechanism is critical in protecting periodontal tissues from hyperreactivity-induced reactions caused by silver nanoparticles. 31 32 33

Moreover, GSH has been implicated in regenerative processes essential for tissue repair, 34 a desirable outcome of bacterial removal facilitated by silver interactions. 33 One of the key regenerative functions of GSH is collagen synthesis, 34 which is crucial for the structural integrity of periodontal tissues. GSH reactivates vitamin C, a cofactor necessary for the hydroxylation of proline and lysine residues on collagen fibrils, leading to the formation of mature and strengthened collagen fibers. 34

Despite the promising theoretical advantages, the practical applications of GSH-stabilized nano-silver have potential drawbacks. Reports indicate an elevation in inflammatory markers, such as interleukins (IL-6 and IL-8) and TNF-α, as observed by Zorraquín-Peña et al. 28 This cytokine response indicates a potential proinflammatory effect of the nanoparticles, which could undermine their therapeutic benefits. 32 Various confounding factors, such as nanoparticle size, duration of administration, and the ratio of GSH and nano-silver particles, may have influenced these outcomes. 33 Although the biochemical synergy between GSH and nano-silver is theoretically promising, its clinical efficacy and safety require extensive investigation. Addressing these factors will be essential for optimizing the therapeutic application of GSH-stabilized silver nanoparticles in the treatment of periodontitis. Therefore, further studies are necessary to comprehensively understand the interactions and effects of GSH-stabilized nano-silver on periodontal therapy.

Similarly, Reise et al 26 explored the potential for Ag ^+^ stability with an alternate carbohydrate complement, where silver–carbohydrate complexes were created through thio-ether binding to silver nitrate. Cytotoxicity was significantly reduced in observed human gingival fibroblasts (HGF), possibly due to the stabilization of AgNO _3_ through a controlled release response via a weaker thio-ether bond. 35 This reduced cytotoxicity underscores the safety and feasibility of these complexes for potential clinical applications. Another intriguing characteristic of increased water solubility was noted in this study, 26 which appeared to promote more widespread distribution of AgNO _3_ . Although not explicitly nano-size, these complexes exhibited relevant antimicrobial efficiency against peri-pathogens, including *F. nucleatum* . Although Reise et al's 26 *in vitro* study model demonstrated the significant antimicrobial efficacy and reduced cytotoxicity of silver(I) carbohydrate complexes, they did not assess their proinflammatory effects. Therefore, further studies are warranted to evaluate the potential proinflammatory responses associated with these compounds. Understanding the inflammatory impact is crucial to ensure their safe and effective application in clinical settings.

The reviewed metallic silver salts, including AgNO _3_ , 38% SDF, and 19% SDF, also demonstrated significant antimicrobial efficacy, notably reducing the viable counts of *F. nucleatum* . The cytotoxic impact of SDF on HGFs is well-established. 32 However, the reviewed studies 24 26 27 did not investigate the safety of silver salts, particularly their cytotoxicity and inflammatory response in periodontal tissues, alongside their antimicrobial efficacy. Numerous studies support the cytotoxicity of HGFs. For instance, in 1998, Hidalgo et al 36 found that silver nitrate exhibited cytotoxicity to cultured fibroblasts at a concentration of 14 × 10 ^−5^ %, with a contact time of 2 hours. In another *in vitro* study, Zhang et al 37 demonstrated that 0.01% SDF was cytotoxic to HGF, with this cytotoxic effect persisting even after 9 weeks of rinsing with artificial saliva. In support of these observations, Ho et al 38 recently reported that SDF toxicity to HGFs was instantaneous and severe. After applying 1 μL of 0.394% SDF, the HGF attachment process was disrupted immediately. Histological evaluations of the treated tissues revealed apoptotic cells in the epithelium and the upper half of the connective tissue. Given the complex structure of periodontal tissues, which include components such as gingival epithelium, connective tissue, and alveolar bone, it is crucial to understand how these tissues respond to cytotoxic agents. 39 Studying the cytotoxicity and inflammatory responses of these tissues is essential, as they play a significant role in the healing of periodontal destruction and supporting bone regeneration. 39 A comprehensive understanding of these responses will help develop safer and more effective treatments that combat microbial pathogens while promoting tissue regeneration and periodontal health.

*Review limitations:* We acknowledge that this review is limited by the relatively small number of studies included in the analysis. The limitation stems primarily from the scarcity of research specifically addressing the antimicrobial efficacy of silver against *Fusobacterium nucleatum.* The purpose of this mini-scoping review was not to provide an exhaustive synthesis but rather to map the existing evidence, identify research gaps, and encourage further investigation. We recognize that the limited scope of current research underscores the need for broader and more diverse investigations in this field, and we hope that our review aims to encourage such advancements.

MIC values ranged from 25 to 500 µg/mL (particle sizes 5–55 nm) ( Table 2 ). Smaller nanoparticles produced stronger antimicrobial activity but also variable cytotoxic thresholds. This needs further research. Another limitation of the studies reviewed is that all were conducted *in vitro* , which may not fully replicate the complex environment of periodontal pockets. The physiological conditions, biofilm structure, and interactions with host tissues *in vivo* can differ significantly from those in laboratory settings. Additionally, the concentrations of silver formulations varied widely across studies, ranging from microgram to milligram levels. These variations could affect the comparability and generalizability of the results. Moreover, the duration of exposure to the silver formulations was not uniform, ranging from 30 minutes to 24 hours, which may have affected the consistency of the results and their applicability in clinical settings.

Fibroblasts were the only mammalian cell type evaluated; no studies included epithelial, osteoblastic, or immune models. Future work should assess these to capture tissue-specific cytotoxicity. Previous studies have also reported the cytotoxic effects of silver formulations on human gingival fibroblasts at specific concentrations. For instance, AgNO _3_ demonstrated cytotoxicity at concentrations exceeding 0.02 mM, and silver diamine fluoride shows cytotoxicity at concentrations as low as 0.01%. However, except for one study, none of the reviewed articles evaluated the inflammatory responses to silver formulations, a crucial factor for understanding their safety and efficacy in periodontal therapy.

### Clinical Relevance

#### Scientific Rationale for the Study

*F. nucleatum* is a key periodontal pathogen contributing to biofilm maturation and chronic inflammation. Silver-based antimicrobials offer an alternative to conventional antibiotics, thereby helping overcome rising resistance.

#### Principal Findings

All reviewed silver formulations showed significant *in vitro* efficacy against *F. nucleatum* . Glutathione-stabilized silver nanoparticles and silver(I) carbohydrate complexes exhibited reduced cytotoxicity. Among all formulations, GSH-AgNPs and Ag(I) carbohydrate complexes exhibited the best efficacy–biocompatibility profile and hold promise for periodontal applications.

#### Practical Implications

Silver-based agents hold promise for adjunctive periodontal therapy. Yet, their safety profiles, especially regarding cytotoxicity and inflammation, require further *in vivo* validation to guide clinical applications.

## Conclusion

The reviewed studies collectively highlight the significant antimicrobial potential of silver-based formulations against *F. nucleatum* , a key periodontal pathogen. Despite promising *in vitro* results, the cytotoxicity and inflammatory responses associated with various silver formulations underscore the necessity for more comprehensive *in vivo* studies. Further research should focus on standardizing the concentration and exposure times of silver formulations, as well as investigating their interactions with host tissues *in vivo* . Additionally, exploring combination therapies could help enhance the therapeutic potential of silver while mitigating its adverse effects. Addressing these challenges will be crucial for translating the antimicrobial properties of silver into effective and safe clinical applications against periodontal pathogens.
